# Radium; Its Use in Some Cases

**Published:** 1910-05

**Authors:** Theodore Eugene Oertel

**Affiliations:** Augusta, Ga.


### RADIUM; ITS USE IN SOME CASES.

BY THEODORE EUGENE OERTEL, M. D., AUGUSTA, GA.

When Mme. Curie and her late lamented husband discovered Radium they opened up a new world of investigation to the physicist and one may say that this discovery and the subsequent train of investigations with radio-active substances is the greatest single forward stride in the progress of the human race. It would seem that Radium first changes into emanations. Then successively into Radium A, B, C, D, E, and F, the latter being in all respects like polonium, which elemental body has recently been separated from pitchblende by Mme. Curie and her co-laborer, M. Debierne. They succeeded in isolating only the tenth part of one milligram which was found to throw off sensible emanations of ozones and helium and to add to the spectrum seven rays which are not detectible in any other known substance. So powerful are its emanations that it rapidly decomposes all chemic and organic substances and split in all directions the quartz vase used as a container. Naturally, the medical world turned with avidity towards this new substance and it was heralded by the over-enthusiastic as a problem panacea for all human ills.

The scarcity of Radium and its high price has rendered it inaccessible to the medical profession at large, but a large number of investigators have been using it therapeutically for a sufficient length of time for us to begin to draw some conclusions as to its merits as a therapeutic agent. Much more time must necessarily elapse before definite conclusions can be drawn, but we are warranted already in the assumption that it will fill a place now vacant in our medical armamentarium.

The best methods of its applications, dosage, and ultimate results are yet to be determined. Often it will succeed where the X-Ray and the Finsen light have failed, and its ready method of application to small surface growths, particularly about the face, eye, and auditory canal, as well as in the mouth and rectum, make it of peculiar value in conditions where often it is next to impossible to use the X-Ray. It is particularly valuable in cases of epithelioma, vascular naevus, eczema with itching, lupus vulgaris, pigmented moles and warts, and cases of cure have been reported on inoperable conditions due to carcinoma and sarcoma so situated that the tube might be buried in their depths..

Relative to the action of Radium on the Tissues, I quote an excerpt from the Journal of March 12th, from the Centralb. f. allg. Path. u. Path. Anat. xx, 243, 1909, reviewing the experiments of C. Guyot, as follows: The experiments of C. Guyot tend to show that the first action of radio-activity is one of stimulation of the vitality of the cells of the epidermis and of the fatty glandular tissues, which leads to a hyperplasia of these tissues. Soon, however, retrogressive processes set in. The entire process is one of acceleration of the normal cycle of cell activity in the epidermis. The normal slow replacement of the superficial cells by new cells from the germinal ephithelium is greatly hastened by the stimulation of the radium rays, and there results an overproduction of cells and a consequent rapid involution in the newly formed cells. Within a short time the germinal ephithelium seems to become exhausted and there follows a halt in the germination of new cells, while the rapid involution of the recently formed cells *and the natural death of the more peripheral ones continue.* This, as is evident, leads to a complete extermination of the epidermis. The muscular tissues seem to be particularly resistant to radio-activity. The experiments were carried out on mice. The animals were placed in a lead-lined satchel in an opening in the lid of which the metal capsule containing the radium was placed. The exposures lasted as long as forty-eight hours and the animals died from three days to seven months after exposure.

I first secured ten milligrams of Radium Bromide of the highest activity in a glass tube, but unfortunately after using this for a period of about six weeks, it met with an accident by which I lost four milligrams, and since then I have employed eleven milligrams of Radium Bromide in an aluminum container. I have found the latter preferable, not only on account of safety, but also for the reason that the alpha rays penetrate the aluminum tube much better than they do glass and the alpha rays are largely in the majority. The purpose of this paper is a preliminary report of a few cases treated with the above medium. Of course, sufficient time has not yet elapsed for us to draw conclusions as to the permanency of the cures in the following cases, they may only be considered as clinical cures. Upon a future occasion, I trust I shall be able to report their final outcome.

*Recurrent Epithelioma of the Nose.* A. B., white male, age 40, consulted me a year ago with epithelioma upon the right side of the nose, nodular in character, two cm. long by 1 cm. wide, and elevated above the surrounding surface with a few puncture ulcerated areas. The growth had been unsuccessfully treated with the X-Ray for a period of almost a year. Under general anesthesia the growth was removed, together with an apparently healthy margin of skin about 1 cm. wide. A linear scar resulted with no apparent recurrence for a period of six months. At this time a small nodule began to grow in the lower border of the scar and within a period of about six weeks was markedly elevated and about 1 cm. in diameter. Three radium exposures were given, extending over a period of three weeks, there being an intermission of one week between each exposure. There resulted marked erythemia over an area of about 2 cm. and the growth rapidly disappeared until at the end of about two weeks further the surface was smooth and apparently healthy and only slight redness remained. At the end of a month's time the skin had assumed a normal hue and appearance.

*Pigmented Hairy Mole of the Face.* Mole was about 7 cm. in diameter by 5 mm. in height. Heavily pigmented and contained numerous dark hairs. Two exposures of one hour each with an interval of one week were given. Marked erythemia with itching and burning resulted and there was also slight vesiculation. The growth rapidly shrunk until it reached the level of the skin and four months later there was only slight elevation. The pigmentation, however, remained. It is probable that a further exposure would remove this also. All of the larger hairs dropped out and there remained only a comparatively few fine ones.

*Pigmented Mole of the Right Upper Lid.* A. R., white male, age 40. The growth was congenital, 5m. wide by 10 mm. long, and involved the cilea. It was extremely vascular and nodular in character, and black in color. A total of five hours exposure was given extending over several weeks. The growth gradually dried up and came off in pieces. At the end of six weeks it had entirely disappeared. Normal skin and lashes remained and there was no contraction of the lid.

*Glandular Carcinoma of the Post-Auricular Glands.* A. J. Q., age 50, white male, consulted me two years ago for ulcerated mass involving lower portion of right lobe of the ear and contiguous tissues, the mass about 3 cm. in diameter by 2 cm. in height, extensively ulcerated with indurated, raised edges. From it he had had several hemorrhages. X-Ray treatment over a considerable period of time reduced the growth but did not eradicate it. Finally, an operation was done under general anesthesia; all unhealthy tissue being apparently removed, the wound being allowed to heal by granulation. Microscopic examination of the tissue showed the growth to be a glandular carcinoma. A number of X-Ray treatments were given after the operation and the patient was then allowed to return to his home in a distant town. Six months after he returned with a recurrence of the growth and a second operation was performed. Post-operative X-Ray exposures were again made and he was urged to return upon the slightest evidence of recurrence. The latter part of November he returned with a nodule 2 cm. long in the post-auricular tissue. Radium exposures were given, amounting to three hours, and he was told to report in two weeks. At this time there was a considerable softening in the growth and a diminution in size. On January 4th, he again reported and I found that the nodule had entirely disappeared and there was no recurrence at other points. The above three exposures were given on successive days, one hour each, and the patient tells me that he had quite a blister at the site of exposure. There was, however, no scar as a result.

*Epithelioma of the Inner Canthus, Left Eye.* J. A. R., white female, age 34. Consulted me for a small epithelioma at the inner canthus of the left eye. Duration one year. Growth was nearly circular, raised above the surface, about 7 mm. in diameter, microscopic examination was not made, but it was typical clinically of ephithelioma. She assured me that it was rapidly increasing in size. Four exposures of radium were given, the first two of one hour each, the second were of one-half hour each, at intervals of one week. The growth rapidly diminished in size and at the end of six weeks from the beginning of treatment had disappeared. So far there has been no recurrence; no scars; and no contraction.

*Itching Eczema of both Auditory Canals.* C. F. T., white male, age 65. Presented himself November 18th, 1909, with a condition of itching and discharging eczema of the auditory canals which had existed for forty years and for which he had been treated in all parts of the world by many specialists of note. There was a foul discharge, intense itching, and burning, and when the canals were wiped clean they were found to be intensely hyperemic, very tender, and bled easily. I introduced the radium tube in each canal for the space of ten minutes as I

did not dare to give a longer exposure without experimentation. The discharge and itching stopped like magic. Treatment has been intermittently continued since that time. Ten exposures have been given, the last one being thirty minutes in duration. The mucous membrane is now of a nearly normal appearance, there being only slight redness; the discharge which ceased upon the first application, has not reappeared; there has been almost no itching; swelling has entirely subsided and the patient experiences a total relief from all of the distressing symptoms which have troubled him for so many years. To me this case is of especial interest on account of its having been entirely intractable to other methods of treatment and yielding so readily to this. The possibility of radium treatment for pathological diseases of the middle ear is one of great interest.

*Keloid of the Breast.* A. B. C., white female, age 25. Suffered a year ago with an abscess of the right breast. In the resulting scar there developed a keloid; 15 mm. in diameter; circular; about 5 mm. in height; pink in color; and of a very dense consistence. A total exposure of ten hours have been given this growth, extending over a period of above three months. At present it is level with the skin, but there is still some induration and it seems certain that a few more treatments will remove it entirely. Treatments have been one hour each, the tube being strapped with adhesive upon the growth, and between exposures there have been intervals of between two to three weeks. There has been considerable erythemia and twice vesiculation at the point of exposure.

*Carcinoma of the Rectum.* F. E. Y., white female, age 56. The case was referred to me November 11, 1909, through the courtesy of Drs. Horne and Crane as an inoperable case of cancer of the rectum in which some good might be attained by the use of radium. Eight years ago the patient began to have rectal trouble with hemorrhages from the bowels and says that at this time she passed two small pieces of bone.

She was operated upon September 14th, 1909, for fistula in anus, at which time a diagnosis of cancer was made. Has lost twenty-five pounds in weight and is quite anemic; has no pain; but is obliged to take cathartics constantly so that her stools may be entirely liquid. Is now taking ten doses of cathartic daily and even with this has much difficulty in defecation. There is an indurated mass in the rectum about 10 cm. from the anus, said mass being anular in form with a small opening admitting a uterine probe with difficulty. The probing causes pain and some bleeding. There is a foul muco-purulent discharge. Exposures were at once instituted; the radium tube being introduced as far as possible into the small central opening mentioned. The radium tube was protected by being enclosed in a small gelatine capsule such as is employed for the protection of thermometers in infectious cases and was held in place with a cotton tampon. Exposures were at first one-half hour, later they have been increased until now I am giving her exposures of an hour at a time. The total of exposures to date is about forty hours.

There seems to have been a considerable amelioration of the distressing symptoms of the case. She has gained eight pounds in weight and after a period of three weeks from the first treatment ceased entirely to take cathartics except occasionally and now has formed actions although the fecal masses are small; about the size of her thumb, she informs me. The central opening of the mass is considerably increased in size so that it is now easy to introduce the tube and it can be pushed through the mass and felt to emerge upon the opposite side. The anular induration seems to be about 5 mm. in width, there has not been any sensible decrease in the discharge and it still has the characteristic odor of cancer of the rectum. Flatulence which troubled the patient greatly before radium treatment was instituted has almost entirely disappeared. She sleeps better and assures me that she feels considerably improved in a general way. As she was rapidly declining at the time that the radium treatment was begun, it would seem as if some good had been accomplished and her days may have been prolonged, at least. It is almost too much to hope for an ultimate cure in such a case, but the patient is very persistent and hopeful, and, of course, I shall keep up the treatment as long as she desires and there is indication for so doing.

Besides the above cases, I have under treatment several others which I trust I shall be able to report favorably upon in a future communication. One case of lupus vulgaris of some years' standing which has been unsuccessfully treated with X-Ray and other medicaments seems to be yielding nicely to radium treatment, but it is too soon for me to make a positive statement in regard to it.

In view of the large number and variety of diseases which have been treated successfully with radium by a host of surgeons in all parts of the world and the successful outcome in the cases above reported, I shall continue to use this marvelous agent with increasing interest.

I thank you for your attention.

